# Novel Hyperspectral Analysis of Thin-Layer Chromatographic Plates—An Application to Fingerprinting of 70 Polish Grasses

**DOI:** 10.3390/molecules28093745

**Published:** 2023-04-26

**Authors:** Joanna Wróbel-Szkolak, Anna Cwener, Łukasz Komsta

**Affiliations:** 1 Department of Medicinal Chemistry, Medical University of Lublin, Jaczewskiego 4, 20-090 Lublin, Poland; 2 Department of Pharmaceutical Botany, Medical University of Lublin, Chodzki 1, 20-093 Lublin, Poland; anna.cwener@umlub.pl; 3 Botanical Garden of Maria Curie-Skłodowska University in Lublin, 3 Sławinkowska Street, 20-810 Lublin, Poland

**Keywords:** hyperspectral photography, high-density range photography, thin-layer chromatography, principal component analysis

## Abstract

The advantages of hyperspectral imaging in videodensitometry are presented and discussed with the example of extracts from 70 Polish grasses. An inexpensive microscope camera was modified to cover the infrared spectrum range, and then 11 combinations of illumination (254 nm, 366 nm, white light), together with various filters (no filter, IRCut, UV, cobalt glass, IR pass), were used to register RGB HDR images of the same plate. It was revealed that the resulting 33 channels of information could be compressed into 5–6 principal components and then visualized separately as grayscale images. We also propose a new approach called principal component artificial coloring of images (PCACI). It allows easy classification of chromatographic spots by presenting three PC components as RGB channels, providing vivid spots with artificial colors and visualizing six principal components on two color images. The infrared region brings additional information to the registered data, orthogonal to the other channels and not redundant with photos in the visible region. This is the first published attempt to use a hyperspectral camera in TLC and it can be clearly concluded that such an approach deserves routine use and further attention.

## 1. Introduction

Image acquisition techniques, together with advanced computer processing, have become a very important approach in recent years in many fields of analytical chemistry [1]. This fact is the result of easy access to high-resolution photography—an average smartphone has photographic abilities that were unreachable for an average scientist even in the relatively recent past [2]. Moreover, the obtained data can be used for comparative analysis of samples with sophisticated mathematical algorithms.

Chemometric fingerprinting [3] is a way to treat the whole signal as a unique pattern without the need to identify features such as chromatographic peaks or spectral maxima. The whole fingerprints are compared with multivariate data mining methods, as each of them can be perceived as a point in high-dimensional space. There is no way to imagine high-dimensional space easily, but it has analogous properties to a plane or three-dimensional space. There is a distance between each pair of points (called Euclidean distance), and any three points (or two points and the origin) form a triangle with specific angles. Chemometric methods are based on dissimilarities between point pairs, or they try to reduce dimensionality by projecting the points to some lower-dimensional subspace.

Regardless of the development of sophisticated instrumental chromatographic techniques, thin-layer chromatography still has its own place in the set of available analytical methods. It is frequently chosen due to low costs, portability of equipment, a very low amount of solvent used for one sample (green approach), as well as the possibility to analyze very complex samples without any extraction (a plate is used once, so there is no need to separate the matrix from the analytes).

There is a long-established practice for obtaining signal-like data from a thin-layer chromatographic plate that was begun even before chemometric algorithms gained popularity [4]. The main aim was then to analyze samples in a quantitative manner by computing the area of the obtained peaks. Densitometers were then used as a main tool because the video-scanning idea was technologically limited. In the 1980s, a video-scanning camera was still a technical novelty [5]. A serious interest began in the 1990s with the availability of special dedicated equipment—an analog color camera connected to a special peripheral component interconnect (PCI) card, which allowed framegrabbing [6]. Another idea to scan a plate with a flatbed scanner was also presented for tasks in which data collection could be conducted in the visible part of the spectrum [7,8,9].

Papers describing videodensitometry in these years focused mainly on quantitative determination of particular compounds and were based only on commercial algorithms implemented in the dedicated software [10,11,12]. Later, when an average computer gained enough memory to perform more advanced image processing, as well the programming languages developed significantly in the meantime, many aspects of the process could be programmed in various ways and optimized [13,14,15], so complete workflows were proposed [16,17]. A serious interest in particular preprocessing steps can also be seen in the literature of that time [18], as well as in combining this approach with many new detection [19] or application methods [20].

The open-source software movement also cannot be neglected in this field of science. Started in the 1980s, it promoted the creation of software that is free in the sense of having no cost (anyone can use it for any purpose) and free in the sense that anyone can improve it and republish an improved version. This provides access to very powerful tools without any costs and allows the modification of software to one’s own requirements by scientists with programming skills. The most important processing tasks can now be performed on images with freely available open-source software [17,21,22].

The main difference between densitometry and video scanning is that the latter is limited to the visible part of the light spectrum. There is a possibility to take a photo in white light as well as in ultraviolet (UV) illumination (254 or 366 nm), where visible fluorescence can be registered. Photographs are taken in three channels, abbreviated as RGB: red, green, and blue visible light, respectively, so there is no possibility to precisely set the wavelength. Photographing the same plate under 254 nm, 366 nm, and visible light yields nine dimensions of information [23,24].

It is also worth emphasizing that video scanning has substantially lower dynamics than densitometry. The standard depth of a digital image is 8-bit, which means that each pixel is stored as three integer numbers in the range of 0–255 for each RGB channel. The exposition settings during acquisition should be carefully chosen, and, in many cases, there can be no possibility of making one shot of a plate while preserving details in dark and light regions. This problem is analogous to classical photography on film, where photosensitivity is also limited by dynamics.

To overcome this problem in photography, a ‘high dynamic range’ (HDR) version was introduced. It is used in artistic photography and the film industry [25]. Our recent research proved that it can also be used successfully in chromatographic video scanning [26]. The idea behind our approach was analogous to other applications of HDR photography: to take several shots of one plate with increasing exposures, then combine them into one image dataset with a huge range of dynamics. By using chemometric analysis it was proved that the HDR approach deals with nonlinearities in dark and bright areas of the plate as well as preserves details in these regions.

A typical camera sensor’s spectral sensitivity is extended towards the infrared range, and this property is widely used in security cameras at night or in artistic infrared photography. Cameras designed for such photography have a special filter that removes the infrared part of the spectrum above 700 nm to eliminate undesired effects. Its removal is relatively easy and technically possible, resulting in a full-spectrum camera. Having in mind that phytochemicals can glow (fluorize) in the infrared region when excited with ultraviolet or visible light, the infrared range seems to be an interesting addition, and one can expect that it would bring new information to a video-scanning result. This inspired us to investigate if this direction of video scanning is worth any effort. It should be emphasized that there were no attempts to photograph TLC plates in a hyperspectral manner, and this is the first attempt reported in the literature.

The second main idea was to investigate whether there is a possibility to compress channels from many images (combinations of illumination and filter) of the same plate with chemometrics and to present the results in several channels only, with artificial coloring.

Our current investigation is related to the fingerprinting of grasses growing in Poland. Surprisingly, there has been no review to date describing the phytochemical composition of the whole family. Only individual subfamilies have been reviewed, including the *Bambusoideae* subfamily [27], *Arundo donax* L. [28], *Cymbopogon* genus [29,30,31], and *Saccharum officinarum* L. [32], as well as South African taxa [33].

The purpose of this idea is to classify the species according to the similarity of their chemical composition and to identify some interesting plants for further, more detailed research in the future. In our previous research [34], we performed fingerprinting with densitometric scanning and visually identified some unknown markers. The identification of them requires further research and mass spectrometry study, as there is no strict literature reference to discuss (only several species were analyzed by this method in the earlier research; for a literature survey, see Table 1 in [34]).

As our second idea was to investigate an idea for infrared extension to video scanning, we realized that grasses are a good group for checking the hyperspectral extension of video scanning. Our previous thin-layer chromatography analysis [34] proved that they contain constituents glowing under ultraviolet light with various colors. Therefore, it can be expected that some spots will also glow in the infrared part of the spectrum, which will, in this case, add new information to the fingerprint.

## 2. Results and Discussion

**Figure 1:**
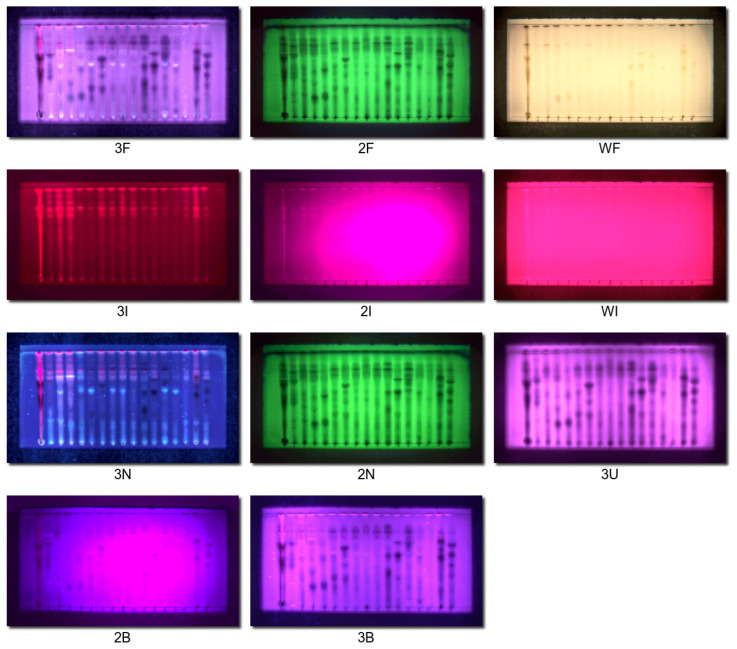
Normalized (with enhanced contrast) photos of TLC plate in 11 studied illumination-filter combinations. For symbol explanation, see Experimental section.

The obtained dataset consisted of 11 images for each plate, acquired using the following combinations of illumination and filters: 2F, 3F, WF, 2I, 3I, WI, 2N, 3N, 2B, 3B, and 3U. Although there are many more combinations, the rest of them did not produce any usable data (for example, it makes no sense to register a blue channel with red illumination, etc.). We have chosen the plate with the first 17 samples for the presentation, as the other plates yielded similar conclusions. Each image contained three colors (RGB: R2F, G2F, B2F, etc.), which yielded 33 channels. Figure 1 presents all registered images with normalized contrast. The 2I and 2B images had very weak spots, which resulted in a visible increase in background drift during contrast stretching. Although this encouraged us, in the first moment, to discard these images from analysis, we discovered that our approach did not force us to do so because PCA very efficiently separates significant information from noise and background artifacts.

### 2.1. Principal Component Analysis

**Figure 2:**
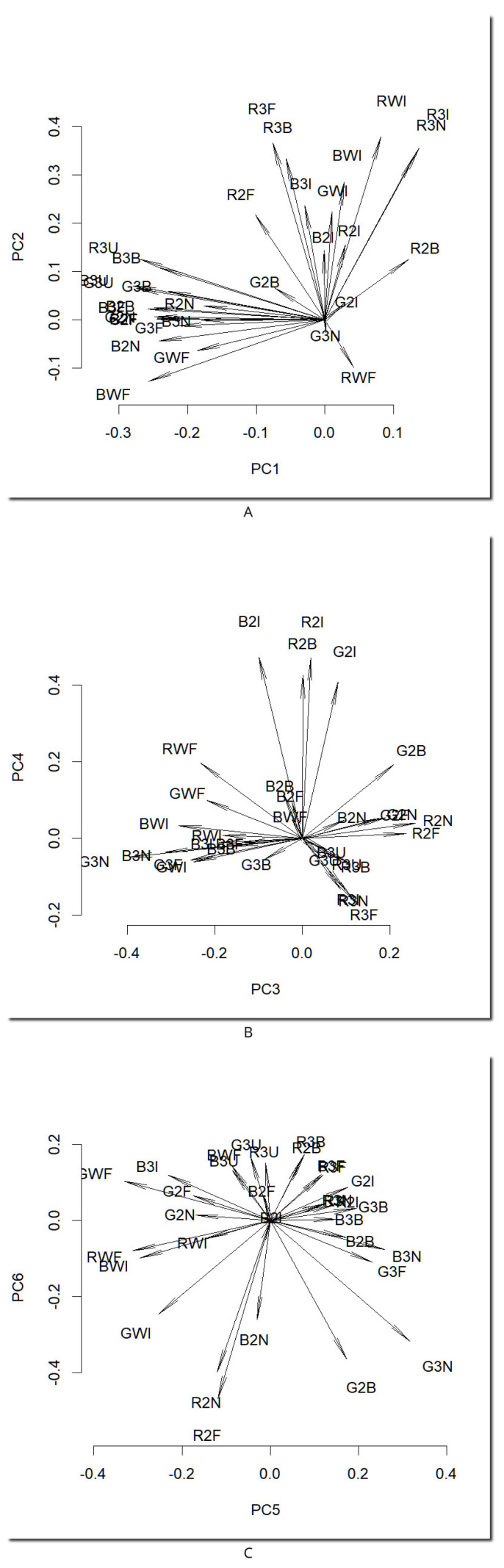
Loadings of the first six principal components of the analyzed dataset: (**A**) PC1 and PC2, (**B**) PC3 and PC4, (**C**) PC5 and PC6. For explanation of symbols, see Experimental section.

**Figure 3:**
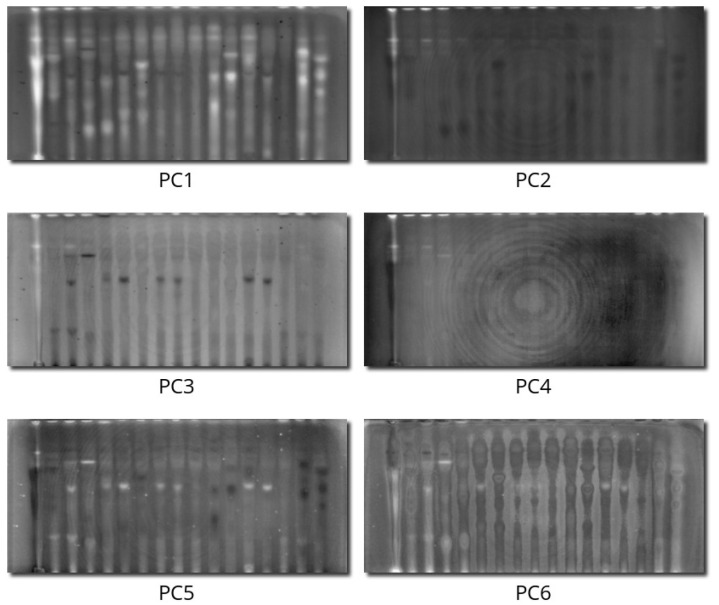
Scores of the first six principal components of the analyzed dataset, presented as grayscale images.

The first six principal components explained 35.4%, 14.9%, 11.9%, 10.2%, 6.7%, and 3.1% of the variance, respectively, which sums to 82.1% of the total information. It can be concluded that a satisfying compression ratio was achieved: 33 intercorrelated hyperspectral channels can be compressed into six channels. Figure 2 presents loadings, whereas Figure 3 presents scores for the first six components.

The first PC, explaining 35.4% of the variance, can be interpreted as an averaged trend among all compounds. The arrows of all channels with a bright background and fluorescence-quenching dark spots are pointing left (Figure 2A). Therefore, this PC contains all the spots that quench the fluorescence and fluorize in the visible spectrum in other modes. Inspecting the scores of this component (Figure 3), it can be seen that, on one side, there is everything not quenching the fluorescence and glowing stronger than the background (several spots and some dust artifacts).

The second PC, explaining 14.9% of the variance, is related to the red fluorescence under 366 nm, which is positively correlated with any fluorescence in the infrared region (arrows pointing upwards in Figure 2A). White spots in the score image (Figure 3) represent spots that glow red both in the visible and infrared regions. The dark spots in this PC are compounds that do not glow at all, and they even quench small background fluorescence under 366 nm (absorbing in this region).

The third PC, explaining 11.9% of the variance, contains differences between cyan fluorescence in 366 nm UV light (G3N, B3N, G3F, and B3F) pointing left in Figure 2B (dark spots in the score image in Figure 3) and red fluorescence under the same illumination (bright spots in the score image).

The fourth PC, explaining 10.2% of the variance, contains information encoded in infrared fluorescence excited only by 254 nm irradiation (arrows pointing upwards in Figure 2B, bright spots in the score image in Figure 3).

The fifth PC, explaining 6.7% of the variance, contains the differences between intensively glowing spots (almost white in 3F, 3N, so containing information in the green channel, bright spots in Figure 3, right-pointing arrows in Figure 2C) and very dark areas, but glowing in infrared (dark spots in the last three samples, dark spots also on the score image).

The sixth PC contains some background drifts, nonlinearity artifacts, and optical distortions, but only small changes in spot intensities; therefore, it can be concluded that all information about hyperspectral spot properties is encoded in the first five PCs.

Concluding, the information in 33 hyperspectral channels can be compressed into six independent trends for further easy analysis and interpretation. It can also be clearly seen that the infrared region brings important non-redundant information to the dataset, so it can be perceived as a significant step ahead in the video scanning technique.

### 2.2. PCACI Results

**Figure 4:**
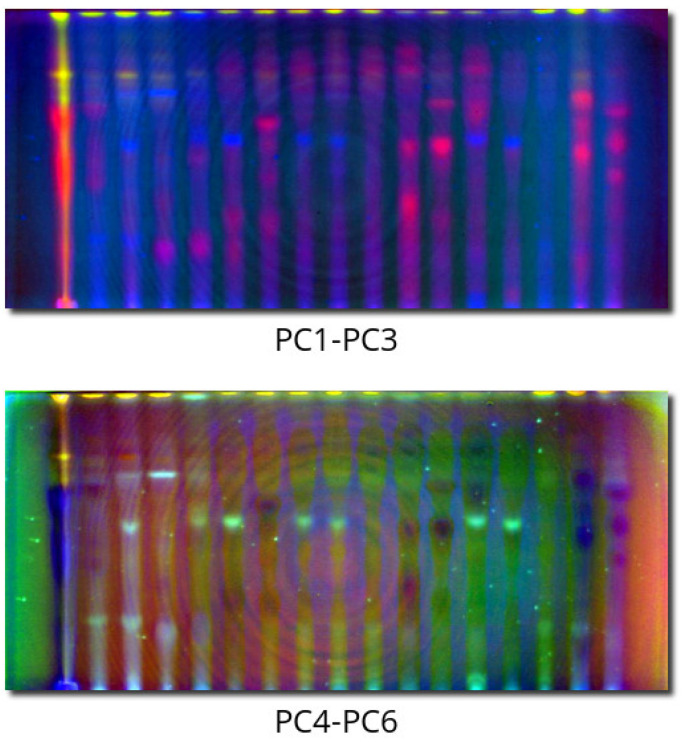
PCACI images for PC1-PC3 and PC4-PC6 principal components.

Figure 4 presents PCACI images of plates with an unchanged order of components. The colors are quite distinct and allow for the classification and grouping of spots according to their hyperspectral properties. The background drift and nonlinearities in the sixth PC do not disturb this classification. PC4-PC6 color provides some complementary information because spots with the same color in PC1-PC3 can differ in color there. Looking at these two PCACI images provides a holistic perspective without the need to compare colors on the original 11 images, preserving the same information. As the color combinations are distinct, they are also a way of classifying spots.

### 2.3. HCA Results

**Figure 5:**
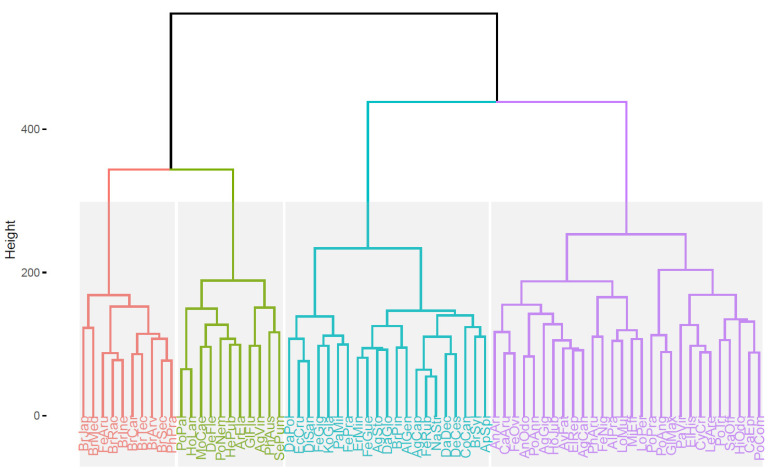
Hierarchical cluster analysis of the fingerprints with optimal division into 4 classes, denoted with distinct colors. Species names are abbreviated in unique manner; for the list see Section 2.

**Figure 6:**
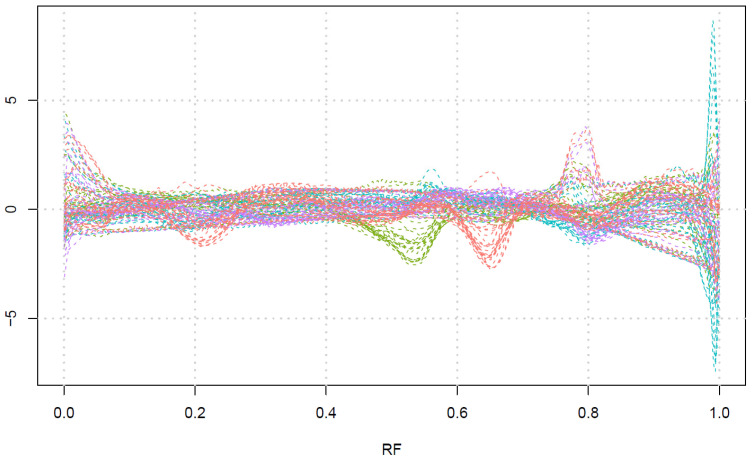
The averaged signal for each class of the sample. Colors are consistent with classes in Figure 5. For each class, 30 channels are plotted separately.

For further fingerprinting analysis, we have arranged the data as a 70 (samples) × 15,000 (pixels) dataset, scaling each signal separately and performing HCA based on the Euclidean distance. Figure 5 presents a dendrogram with optimal division of the grass species into four classes. Figure 6 gathers the averaged fingerprints of all 30 channels for each of the four classes. Investigating these fingerprints, one can conclude that the clustering is performed in the following way:

1. The red class corresponds to the presence of a band around RF 0.2 and around RF 0.65, with or without a band around RF 0.8.
2. The green class corresponds to the presence of a band around RF 0.55.
3. The cyan class corresponds to the presence of strong bands glowing red or infrared at the end of the plate (RF close to 1), with very weak bands in other parts of the plate.
4. The magenta class corresponds to the bands mainly around RF 0.8.

Comparing this clustering to our previous work [34], where we divided the grasses into 5 groups by densitometric fingerprints, one can conclude that the clustering is performed in a generally similar way. The magenta class corresponds to classes 4 and 5 from the previous paper; the red class is mostly consistent with the earlier class 3, while green and cyan corresponds to class 1 and 2, respectively. However, the intense peaks at the end of the plate in the cyan class referred to infrared fluorescence, which is absent in our earlier work and proves that hyperspectral photography gathers more information than densitometry.

## 3. Materials and Methods

### 3.1. Plant Material

Almost all grasses (except two noted further) used in the study were collected from natural stands and habitats by Joanna Wróbel-Szkolak during the 2020 season in Poland. Species were determined, according to the current literature, by Anna Cwener. Herbarium specimens containing dates, locations, and further information are available for future reference. Panicum virgatum ‘Shanendoah’ plant material was kindly donated by Meadowlark Botanical Garden (Vienna, VA, USA). Saccharum officinarum was cultivated in the botanical garden of Maria Curie-Skłodowska University in Lublin, Poland.

The investigated grasses were arranged as follows: (1) *Agrostis canina* L.; (2) *Agrostis capillaris* L.; (3) *Agrostis gigantea* Roth; (4) *Agrostis stolonifera* L.; (5) *Agrostis vinealis* Schreb.; (6) *Alopecurus geniculatus* L.; (7) *Alopecurus pratensis* L.; (8) *Anthoxanthum aristatum* Boiss.; (9) *Anthoxanthum odoratum* L.; (10) *Apera spica-venti* (L.) P.Beauv.; (11) *Arrhenatherum elatius* (L.) P.Beauv. ex J.Presl i C.Presl.; (12) *Avena fatua* L.; (13) *Brachypodium pinnatum* (L.) P.Beauv.; (14) *Brachypodium sylvaticum* (Huds.) P.Beauv.; (15) *Briza media* L.; (16) *Bromus arvensis* L.; (17) *Bromus carinatus* Haczyk. and Arn.; (18) *Bromus inermis* Leyss.; (19) *Bromus japonicus* Thunb.; (20) *Bromus racemosus* L.; (21) *Bromus secalinus* L.; (22) *Bromus tectorum* L.; (23) *Calamagrostis arundinacea* (L.) Roth; (24) *Calamagrostis epigejos* (L.) Roth; (25) *Corynephorus canescens* (L.) P.Beauv.; (26) *Cynosurus cristatus* L.; (27) *Dactylis glomerata* L.; (28) *Dactylis polygama* Horv.; (29) *Danthonia decumbens* (L.) DC.; (30) *Deschampsia cespitosa* (L.) P. Beauv.; (31) *Deschampsia flexuosa* (L.) Trin.; (32) *Digitaria sanguinalis* (L.) Scop.; (33) *Echinochloa crus-galli* (L.) P. Beauv.; (34) *Elymus hispidus* (Opiz) Melderis; (35) *Elymus repens* (L.) Gould; (36) *Eragrostis minor* Host; (37) *Festuca arundinacea* Schreb.; (38) *Festuca gigantea* (L.) Vill.; (39) *Festuca guestphalica* Boenn. ex Rchb.; (40) *Festuca nigrescens* Lam.; (41) *Festuca ovina* L.; (42) *Festuca pratensis* Huds.; (43) *Festuca rubra* L.; (44) *Glyceria fluitans* (L.) R.Br.; (45) *Glyceria maxima* (Hartm.) Holmb.; (46) *Helictotrichon pubescens* (Huds.) Schult. and Schult.f.; (47) *Hierochloe odorata* (L.) P. Beauv.; (48) *Holcus lanatus* L.; (49) *Hordeum jubatum* L.; (50) *Koeleria glauca* (Spreng.) DC.; (51) *Leymus arenarius* (L.) Hochst.; (52) *Lolium multiflorum* Lam.; (53) *Lolium perenne* L.; (54) *Milium effusum* L.; (55) *Molinia caerulea* (L.) Moench; (56) *Nardus stricta* L.; (57) *Panicum miliaceum* L.; (58) *Panicum virgatum* ‘Shenandoah’; (59) *Phalaris arundinacea* L.; (60) *Phleum pratense* L.; (61) *Phragmites australis* (Cav.) Trin. ex Steud.; (62) *Poa angustifolia* L.; (63) *Poa annua* L.; (64) *Poa compressa* L.; (65) *Poa nemoralis* L.; (66) *Poa palustris* L.; (67) *Poa pratensis* L.; (68) *Poa trivialis* L.; (69) *Saccharum officinarum* L.; and (70) *Setaria pumila* (Poir.) Roem. and Schult.

### 3.2. Sample Preparation

The dried whole-plant material was carefully crushed with a mechanical grinder, and 2.5 g of weighted sample was extracted three times with 25 mL of a methanol–acetone–water (3:1:1) mixture. Each time, the mixture was extracted using 15 min of ultrasonication at 35 °C. The extracts were joined together in a round-bottomed flask and evaporated to dryness in 35–40 °C in a vacuum with a rotary evaporator. Each dried extract was dissolved in 5 mL of methanol and used for further analysis.

### 3.3. Chromatographic Conditions

Horizontal thin-layer chromatography chambers were used for all experiments (Chromdes, Lublin, Poland). A volume of 5 μL of extract was applied 5 mm from the plate’s edge with a microsyringe (Hamilton, Bonaduz, Switzerland). After evaporation of the solvent during application, plates were developed with ethyl acetate–methanol–water (8:2:2) in sandwich mode (without saturation, stationary phase upwards) to 5 mm from the opposite edge (90 mm of development distance), then dried at ambient temperature and immediately processed as described further. All experiments were carried out in an air-conditioned room with the temperature set to 23 °C.

### 3.4. Image Acquisition Procedure

An inexpensive no-name 5-megapixel microscope camera with USB 2.0 interface (producer unknown, sold online by many sellers) was modified by disassembling and carefully removing the internal IR-cut filter (therefore converting it to a full-spectrum camera). It was mounted on the top of a Desaga (Wiesloch, Germany) CabUVIS video-scanning chamber, equipped with original white (denoted as **W**), 254 (**2**) and 366 nm (**3**) fluorescent tubes. To enhance the possibility of illumination, an additional RGB-led strip was added, which allowed for visual illumination in three separate parts of the spectrum (**R**, **G**, and **B**, respectively). A D-Max DW-27125DIR lens, originally dedicated to monitoring surveillance cameras, was used instead of a microscope adapter. It can be attached to the camera without any adapters, as the C-mount type of interface is the same as on surveillance cameras.

We have used various combinations of illumination and filtering. Images were registered without any filter (full spectrum, denoted as **F**), with a UV-IR cut filter dedicated to a telescope (denoted as **N**, it is equivalent to an unmodified camera), a Hoya R72 infrared-pass filter (**I**), a UV-pass ZB2 filter (**U**), and a cobalt glass plate (**B**). We registered the following combinations of illuminations and filters: 2F, 3F, WF, 2I, 3I, WI, 2N, 3N, 2B, 3B, and 3U. Other combinations did not produce any visible spots.

For each combination, we used the novel acquisition approach developed in our laboratory and previously described [26]. Nine exposures in one exposure value step (EV) were taken and converted to a high-density range (HDR) image with 1280 × 720 pixel resolution. An in-house script written in Python was used to grab the images automatically and to assemble them into one HDR image with algorithms available in the OpenCV library. Raw image data were then transferred to the GNU R environment under R Studio. As all images were taken in color mode, it resulted in 1280 × 720 × 3 tensor for each image (RGB color intensities are further abbreviated as R2F, G2F, B2F, etc.). After visual inspection, we cropped the image to contain only the pixels of the chromatographic plate that resulted in an 1105 × 500 dimension of each image.

For comparative analysis, we extracted tracks (15 pixels wide with averaging) for each sample and arranged them as 70 (samples) × 500 (pixels) × 30 (channels) tensor. Each signal was independently standardized to have a zero mean and unit variance.

### 3.5. Principal Component Analysis

Principal component analysis (PCA) is a basic and popular tool used in the analysis and interpretation of multivariate data. The original dataset is converted to a set of new variables called principal components (scores), which are linear combinations of the original ones. The process preserves as much information as possible in the first scores; therefore, it can be understood as a data compression method. Moreover, the principal components are not intercorrelated at all, so information in subsequent principal components never repeats (there is no redundant information). Therefore, the information encoded in many channels (combinations of illumination, filter, and registered color) can be reduced to several orthogonal channels.

The PCA processing can be perceived as a rotation of the coordinate system in the multivariate space. There are an infinite number of possible rotations, but PCA finds the only possible rotation that preserves the assumptions of this method (compression and orthogonality).

In our approach, PCA was performed on a very tall matrix containing, in each column, image pixels unfolded from a matrix (image) to a vector. Therefore, the analyzed matrix contained 1105 × 500 = 552,500 rows (pixels) and 33 columns (channels). Due to large differences in absolute light intensities, a scaled PCA (to unit variance) was used. An unscaled version would extract information only from the most intensive channels, whereas a scaled version treats all channels equally.

### 3.6. Principal Component Artificial Colouring of Images (PCACI)

PCACI is a new method introduced and proposed in this paper to visualize TLC plates as colorful spots with artificial colors on a dark background. The algorithm of this approach works as follows:

1. Each channel of an acquired image (for example, red color under UV illumination, green color under visible light, red color with an infrared filter) is unfolded from the matrix into a large long column vector.
2. All column vectors are combined into one matrix with many rows (number of pixels) and a column number equivalent to the number of all processing channels.
3. Scaled PCA is done on such a matrix, resulting in matrix scores of the same dimension (as the matrix is tall, the number of principal components is exactly the same as the original dimensionality).
4. Three principal components are then chosen. Most frequently, the best result is achieved with the first three, but one should not be afraid of trying other combinations. The order of the chosen components (assignment to red, green, and blue, respectively) can also be set according to color preferences or a need to emphasize something with a desired color.
5. The score values are scaled to be integer numbers in the range of 0–255 (the pixel with the minimal score becomes zero, the maximal-value pixel becomes 255), representing the intensity of one of the RGB colors in the image.
6. To provide the consistency of the dark background idea, the median of the intensities is computed and checked. If it is larger than 128, a negative is taken to darken the image.
7. The created artificial image is then stored in a common graphical format.

PCACI provides the possibility to present three PCs on one image with easy visual adaptation and vivid colors for the spots. It is much easier to analyze than three separate grayscale images presenting each of the components separately.

### 3.7. Hierarchical Cluster Analysis (HCA)

Hierarchical cluster analysis (HCA) is a popular technique to visualize differences and similarities between objects in multivariate space based on an arbitrary dissimilarity measure. The most common measure is the Euclidean distance between objects, and this measure was used throughout this paper. The result of the analysis is called a dendrogram. It allows easy visual interpretation and the choice of an optimal number of clusters for classification. We have performed analysis on a 70 (samples) x 15,000 (pixels) matrix made by unfolding the 70 × 500 × 30 matrix.

## 4. Conclusions

This paper presents the first trial of hyperspectral photography of TLC plates, extending the gathered information to fluorescence in the infrared region. By analyzing the results, one can summarize this investigation as follows:

1. The addition of infrared channels to video scanning is not redundant and can help to register which spots can glow in the infrared region when excited by UV or visible light. It can record phenomena that cannot be caught by visible-light photography or by densitometric scanning of the plate, as in our previous study.
2. When PCA is performed on the hyperspectral data, the information in the infrared channels is encoded in one particular PC. It proves that this information is orthogonal to the other channels and increases the information in the whole dataset.
3. Principal component analysis can reduce many channels to several (six in our case) orthogonal “pseudochannels”, which are uncorrelated and not redundant. Presenting them as artificial colors (PCACI) allows easy visualization and classification of spots in TLC fingerprints of complex mixtures.
4. Hyperspectral photography is as effective as multi-mode densitometry; moreover, it contains infrared channels, which cannot be registered by a densitometer.
5. There is a need for further research on the topic and hyperspectral photography seems to be an approach deserving of routine use in TLC fingerprinting.

## Data Availability

Data are available from authors upon request.
